# Symptomatic visceral arterial thrombosis in a patient with polycythemia vera successfully treated with mesenteric revascularization: A case report

**DOI:** 10.1097/MD.0000000000044346

**Published:** 2025-09-05

**Authors:** Yohei Yamamoto, Kazuki Tsukuda, Ai Kazama, Yoshiki Wada, Toru Kikuchi, Tsuyoshi Ichinose, Toshifumi Kudo

**Affiliations:** a Division of Vascular Surgery, Department of Cardiovascular Surgery, Institute of Science Tokyo, Tokyo, Japan.

**Keywords:** celiac artery, chronic mesenteric ischemia, polycythemia vera, revascularization, superior mesenteric artery

## Abstract

**Rationale:**

Polycythemia vera (PV) is a type of myeloproliferative disorder, and thrombosis is one of its important complications. Arterial thrombosis commonly occurs in the coronary and cerebral arteries; however, reports of thrombosis in other arteries are limited, and it is even rarer in visceral arteries.

**Patient concerns:**

A 50-year-old woman with PV presented with anorexia and epigastric pain. Detailed history taking revealed a 2-year history of postprandial abdominal pain.

**Diagnoses:**

Enhanced computed tomography revealed severe stenosis of the celiac artery and thrombotic occlusion of the common hepatic, splenic, and superior mesenteric arteries. The patient was diagnosed with chronic mesenteric ischemia.

**Interventions:**

The patient underwent an infrarenal aorta-to-superior mesenteric artery bypass using a great saphenous vein graft.

**Outcomes:**

The postoperative course was uneventful, and her gastrointestinal symptoms resolved completely.

**Lessons:**

The present case highlights that a patient with PV can develop symptomatic visceral arterial thrombosis. Open mesenteric bypass surgery effectively relieved symptoms.

## 1. Introduction

Polycythemia vera (PV) is a disorder characterized by an overproduction of red blood cells, and thromboembolic events are the most important complication in patients with PV.^[1]^

Arterial thrombosis commonly occurs in the coronary and cerebral arteries; however, reports of thrombosis in other arteries are limited, and it is even rarer in visceral arteries. Herein, we report a case of PV in a patient who developed severe chronic mesenteric ischemia (CMI) due to thrombotic occlusion of the celiac branches and the superior mesenteric artery (SMA). The patient provided written informed consent to the report of her case details and imaging studies. Ethics approval was not required for this case report in accordance with the institutional guidelines.

## 2. Case report

A 50-year-old woman presented with anorexia and epigastric pain. Detailed history taking revealed that she had a 2-year history of postprandial abdominal pain, which worsened 2 months ago. She also had a smoking habit of 10 cigarettes per day for over 20 years. Her medical history was notable for PV, diagnosed 9 years prior to this presentation following investigations of an abnormal complete blood cell count. At the time of diagnosis, her complete blood count results showed a white blood cell count of 19.5 × 10^9^/L, a hemoglobin level of 16.9 g/dL, a hematocrit of 50.7%, and a platelet count of 522 × 10^9^/L. Genetic testing confirmed the presence of the JAK2V617F mutation. For several years leading up to this presentation, she was treated with ruxolitinib, occasional phlebotomy, and clopidogrel, and her red blood cell levels were well controlled. In this case, clopidogrel was prescribed instead of aspirin because the treating physician was concerned that aspirin might aggravate her postprandial abdominal pain. Apart from PV, she had no other significant medical history or underlying conditions. The patient’s weight was 41.7 kg, height 160 cm, and body mass index 16.3. On physical examination, her left femoral pulse was absent. Laboratory findings on admission showed a white blood cell count of 9.0 × 10^9^/L, a hemoglobin level of 11.4 g/dL, a hematocrit of 36.6%, and a platelet count of 259 × 10^9^/L.

**Figure 1.**
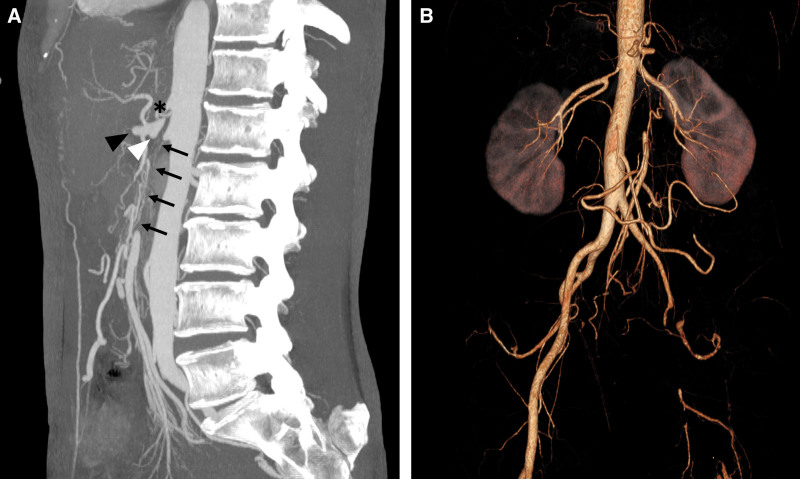
Computed tomography images showing severe stenosis of the celiac artery (asterisk) and thrombotic occlusion of the common hepatic artery (white arrowhead), splenic artery (black arrowhead), and superior mesenteric artery (arrows). (A) Sagittal image. (B) 3-Dimensional reconstruction image.

**Figure 2.**
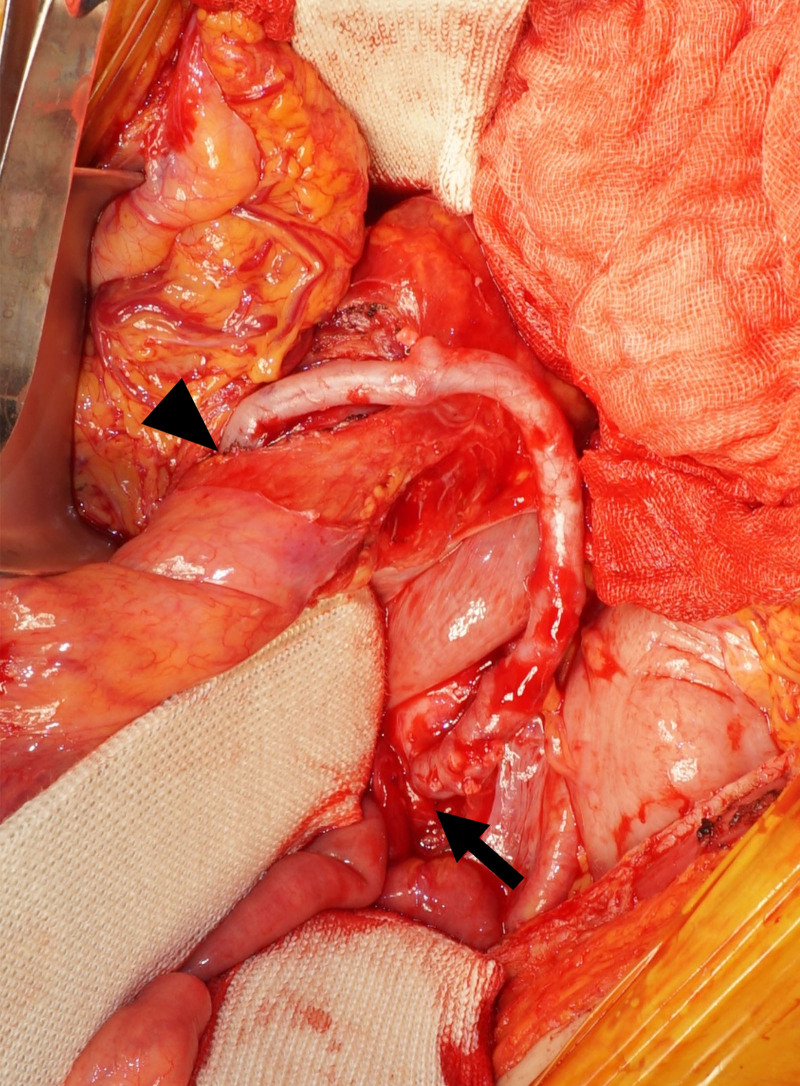
Intraoperative image showing the aorta (arrow) to superior mesenteric artery (arrowhead) bypass using a great saphenous vein graft.

**Figure 3.**
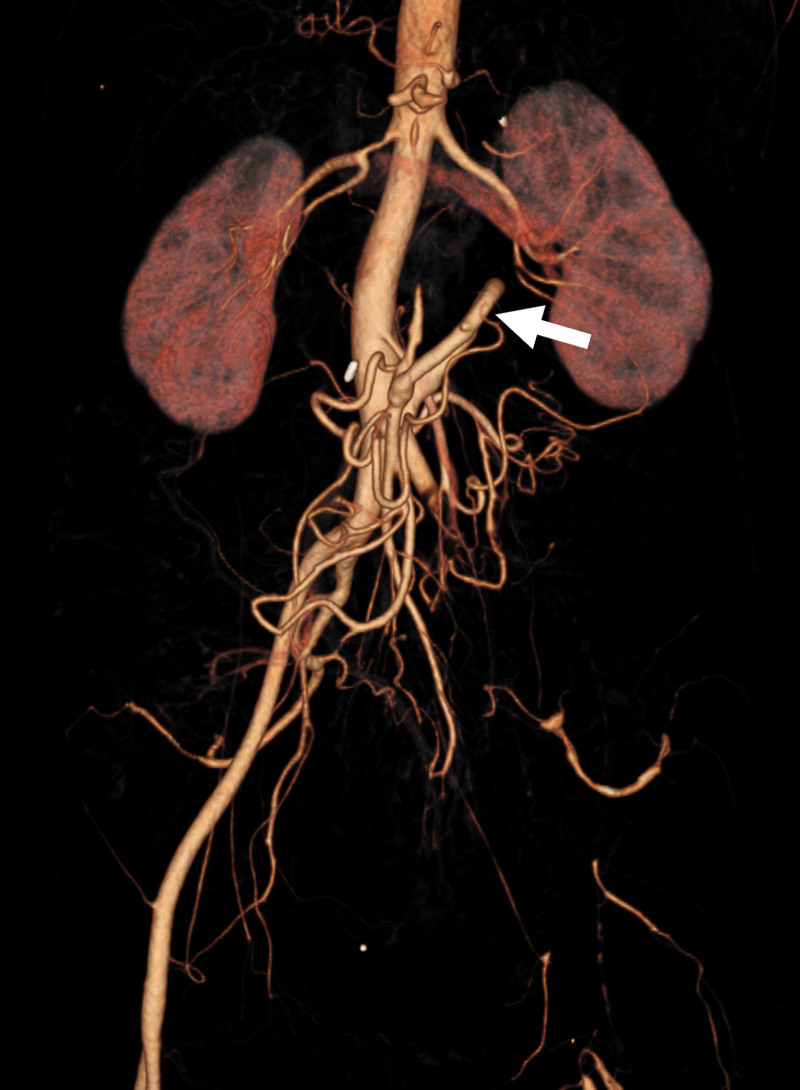
Postoperative computed tomography showing enhanced mesenteric blood flow through the bypass graft (arrow).

An upper gastrointestinal endoscopy was performed, revealing mucosal damage in the stomach suggestive of ischemic gastritis. Enhanced computed tomography revealed severe stenosis of the celiac artery and thrombotic occlusion of the common hepatic artery, splenic artery, and SMA. It also revealed occlusion of the left external iliac artery (Fig. 1). After admission, the patient was treated with parenteral nutrition. She was referred to our department with the diagnosis of severe CMI. Given the severity of her symptoms and the long-segment occlusion involving the SMA, surgical revascularization was considered more suitable than endovascular approach. Therefore, open bypass surgery was selected. The patient underwent an infrarenal aorta-to-SMA bypass using a great saphenous vein graft (Fig. 2). The postoperative course was uneventful, and she was discharged 14 days after surgery. Postoperative computed tomography demonstrated enhanced mesenteric blood flow through the bypass graft (Fig. 3). Her gastrointestinal symptoms completely resolved postoperatively, and she gained 9 kg within 6 months after the operation, indicating an improvement in her nutritional status. The patient is currently under consideration for revascularization to treat the occlusion of the left external iliac artery.

## 3. Discussion

PV is a subtype of myeloproliferative neoplasms, characterized by an overproduction of red blood cells.^[2]^ As demonstrated in the present case, almost all patients with PV have JAK2 V617F mutations.

Thrombosis is the most important complication in patients with PV,^[1]^ and ~25% of patients experience thrombotic events before or at the diagnosis of PV.^[3,4]^ The incidence of thromboembolic complications after the diagnosis of PV varies depending on the literature. Barbui et al^[5]^ reported that the incidence of arterial and venous thrombosis after diagnosis of PV was 2.6% per year. Pemmaraju et al^[6]^ investigated Medicare beneficiaries with PV and reported that 28.4% of patients experienced a thrombotic event at a median follow-up of 34.5 months after diagnosis. Our patient developed postprandial abdominal pain, suggesting the onset of visceral arterial thrombosis ~7 years after the diagnosis of PV.

According to recent studies, the coronary and cerebral arteries are the most common sites of arterial thrombosis in patients with PV, whereas deep vein thrombosis with subsequent pulmonary embolism and visceral venous thrombosis are among the most common venous events.^[1,5,6]^

Regarding the cause of death in patients with PV, progression to myelofibrosis or leukemia accounts for ~15% of cases; however, cardiovascular causes are more common, accounting for 40% of cases.^[7,8]^ Therefore, the recognition of thrombotic events in patients with PV is crucial for achieving good outcomes. Although arterial thrombosis frequently occurs in the coronary and cerebral arteries, its incidence in the visceral arteries is rare and has been reported only in a few case studies.

In 1979, Cryer and Kissane^[9]^ reported a case of PV resulting in death due to infarction of nearly the entire small intestine. An autopsy revealed a thrombus occluding the SMA, as well as numerous small thrombi in small veins across multiple organs, indicating disseminated intravascular coagulation. A case of PV complicated by ischemic enteritis with localized intestinal stenosis, caused by mesenteric venous thrombosis and subsequent thrombus formation in microarteries within the intestinal wall, has also been reported.^[10]^

Although the detailed mechanisms of thrombophilia in PV are complex, blood stasis in the microvasculature due to increased blood viscosity plays a significant role in the development of thrombotic events in patients with PV.^[11]^

What is unusual in this case is that thrombosis occurred in the proximal or mid portion of the visceral arteries and the iliac artery, while the distal segments remained patent. We speculate that thrombotic occlusion of the common hepatic artery and splenic artery may have been associated with reduced blood flow caused by celiac artery stenosis, probably due to median arcuate ligament compression. However, the SMA and the external iliac artery occluded without an apparent anatomical cause.

Her smoking habit or the use of clopidogrel instead of aspirin may have contributed to the development of thrombosis. Smoking is a well-established risk factor that promotes a prothrombotic state^[12]^ and may have compounded her underlying thrombophilia associated with PV. There is no evidence that clopidogrel is effective in preventing thrombotic events in patients with PV.^[13]^ It is also possible that undetected underlying causes contributed to these thrombotic occlusions.

CMI is a medical condition usually associated with stenosis or occlusion of the celiac artery or the SMA. Generally, ischemic symptoms occur when at least 2 of the 3 visceral arterial branches are stenosed or occluded.

In the present case, in addition to the occlusion of the proximal SMA, vascular communication between the common hepatic artery and splenic artery was also disrupted. Therefore, our patient exhibited severe chronic, rather than acute, ischemic symptoms.

In symptomatic patients with CMI, revascularization is indicated. Recently, less invasive endovascular treatment has been recommended as the first-line treatment. Open bypass surgery, however, offers durable patency and better symptomatic relief.^[14]^ In our patient, the occlusion involved both the SMA and celiac branches and the SMA, which was targeted for revascularization, had a long-segment occlusion, making endovascular revascularization technically challenging and less likely to achieve sufficient symptom relief. To the best of our knowledge, this is the first reported case of PV in which mesenteric revascularization was performed.

Surgical treatment in patients with PV can be complicated by both perioperative thrombotic and paradoxical bleeding events. In a multicenter study, the rates of postoperative thrombotic and major bleeding complications were reported to be 7.7% and 7.3%, respectively.^[15]^ These findings highlight the importance of careful perioperative management, including thromboprophylaxis, in PV patients undergoing surgery. In the present case, routine intraoperative systemic heparinization was employed during arterial clamping, with the activated clotting time maintained above 200 seconds. The procedure was completed without complications.

## 4. Conclusions

The present case highlights that symptomatic visceral arterial thrombosis can occur in patients with PV, although it is very rare. Open mesenteric bypass surgery effectively relieved symptoms and may be a viable treatment option in selected cases.

## Author contributions

**Conceptualization:** Yohei Yamamoto, Kazuki Tsukuda, Ai Kazama, Yoshiki Wada, Toru Kikuchi, Tsuyoshi Ichinose, Toshifumi Kudo.

**Resources:** Yohei Yamamoto, Kazuki Tsukuda, Ai Kazama, Yoshiki Wada, Toru Kikuchi, Tsuyoshi Ichinose.

**Supervision:** Toshifumi Kudo.

**Writing – original draft:** Yohei Yamamoto.

**Writing – review & editing:** Kazuki Tsukuda, Ai Kazama, Yoshiki Wada, Toru Kikuchi, Tsuyoshi Ichinose, Toshifumi Kudo.
